# Supplementary material for: Healthy cloned offspring derived from freeze-dried somatic cells
Source: Nat Commun. 2022 Jul 5;13:3666. doi: 10.1038/s41467-022-31216-4 (PMC9256722; doi:10.1038/s41467-022-31216-4)
Supplement: Supplementary file 1 — Supplementary Information [file 41467_2022_31216_MOESM1_ESM.pdf]

# Supplementary information

## Healthy cloned offspring derived from freeze-dried somatic cells

Sayaka Wakayama[1,2,*], Daiyu Ito[1], Erika Hayashi[1], Takashi Ishiuchi[1], and Teruhiko Wakayama[1,2,*]

[1)] *Faculty of Life and Environmental Science, University of Yamanashi, Kofu, 400-8510, Japan*

[2)] *Advanced Biotechnology Center, University of Yamanashi, Kofu, 400-8510, Japan*

*To whom correspondence should be addressed.

E-mail: sayakaw@yamanashi.ac.jp and twakayama@yamanashi.ac.jp

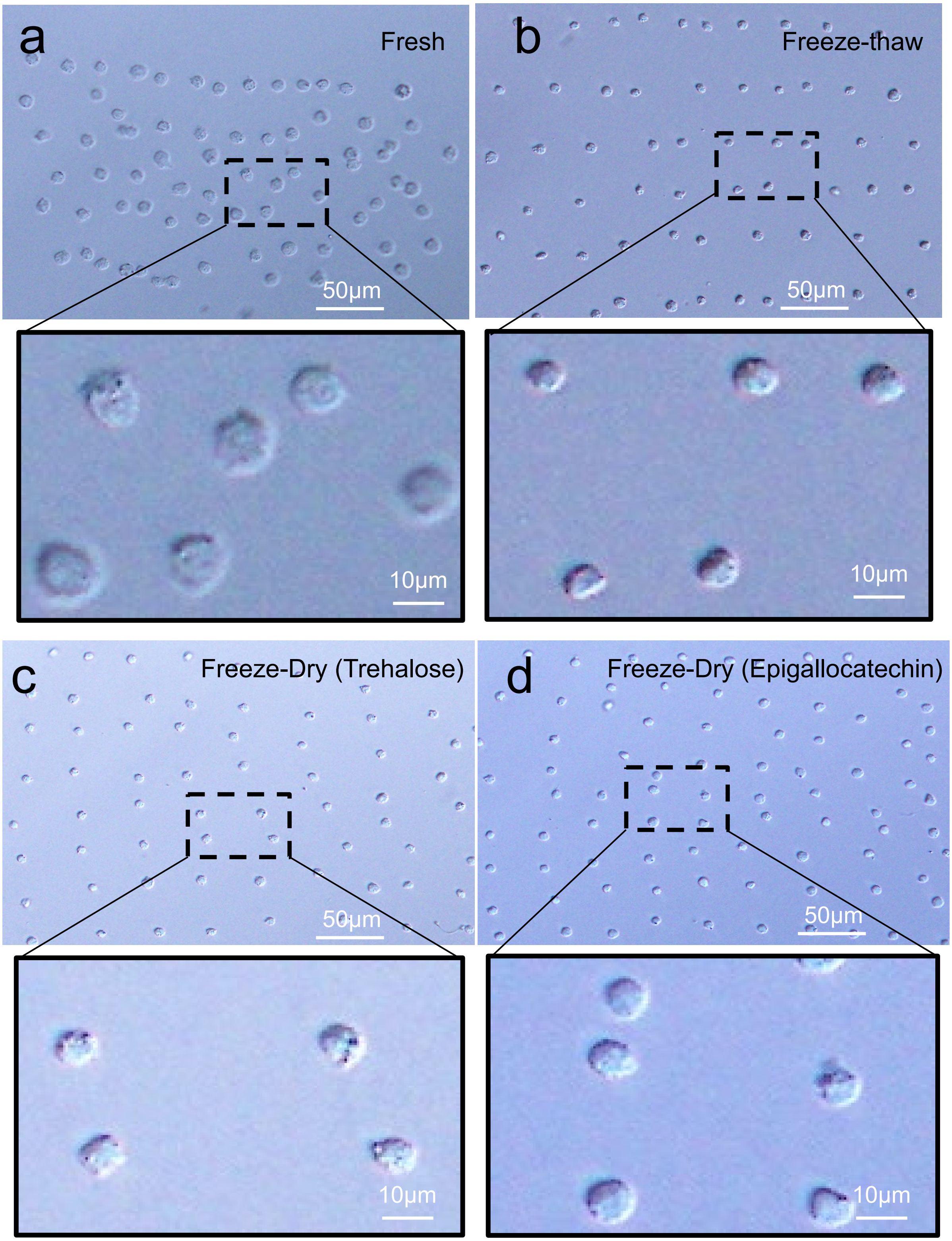


**Supplementary Fig. 1. Morphology of rehydrated FD somatic.**
**a**, Fresh cumulus cells immediately after collection. **b**, Cumulus cells were frozen at –30 °C for more than one week and observed immediately after thawing. **c, d**, Cumulus cells were treated with trehalose **c** or epigallocatechin **d** before FD treatment and observed immediately after rehydration. The cells were put into PVP medium, collected by micro pipetting, and lined up for imaging. More than 20 ampoules in each experiments were observed during nuclear transfer..

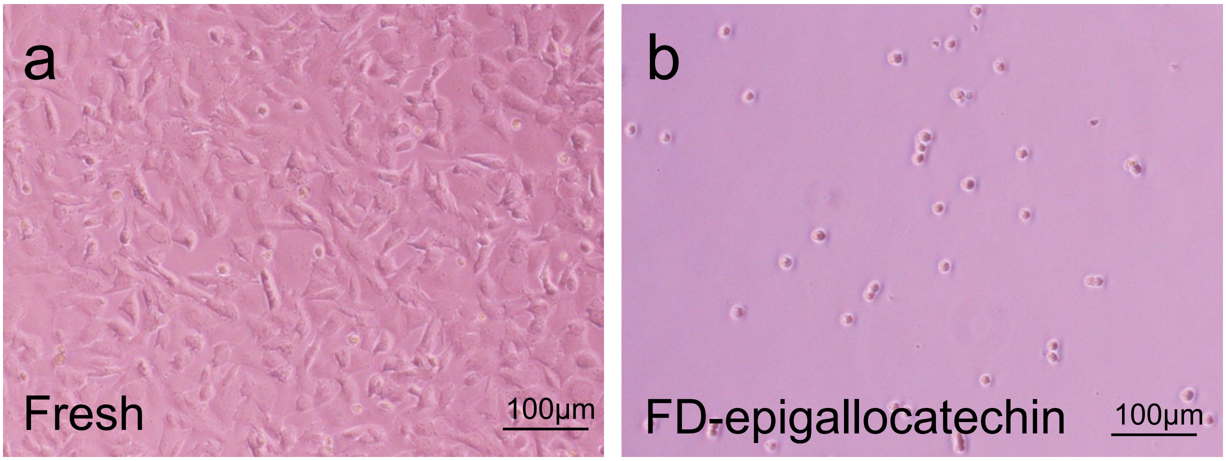


**Supplementary Fig. 2. FD cumulus cell cultures after rehydration.**

**a,** Fresh cumulus cells attached onto the plate the next day. **b,** FD cumulus cells that did not attach onto the plate even when cultured for more than one week. n=4 biologically independent ampoules.

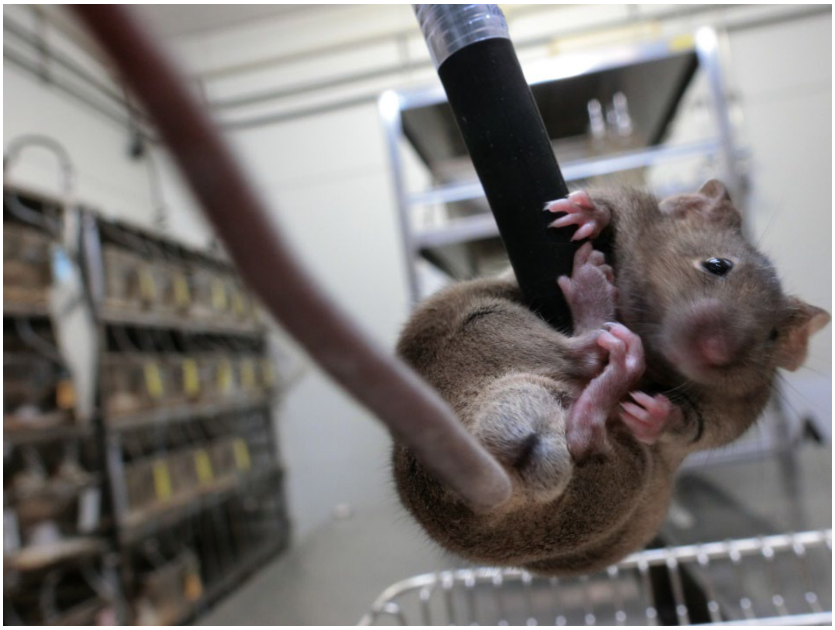

**Supplementary Fig. 3. Cloned male mouse derived from FD fibroblasts of BCF1.**
The cloned male mouse had agouti coat colour, similar to the donor mouse.

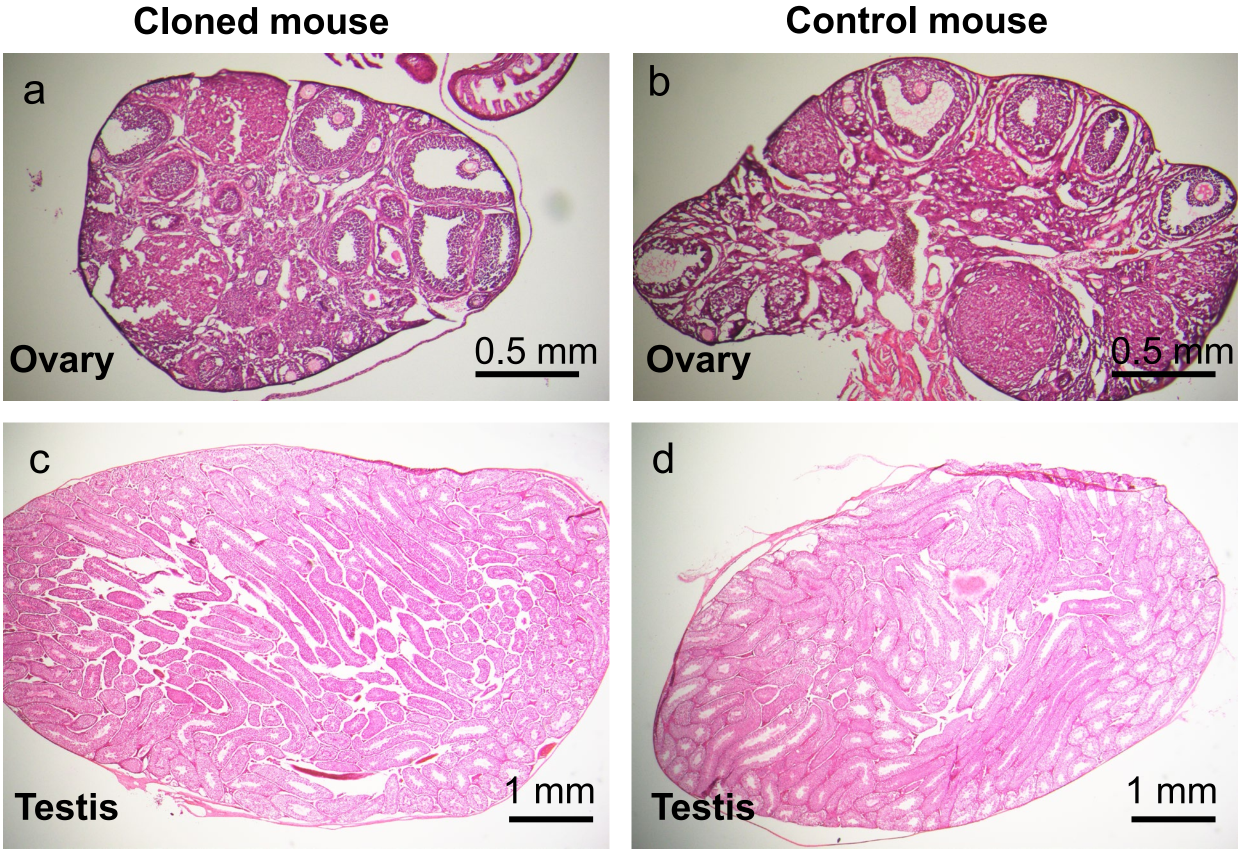


**Supplementary Fig. 4. Histological analysis of ovary and testis of cloned mice**
Stereomicroscopic picture of ovary from a cloned mouse derived from a male donor.
**a,** Several growing follicles, including a Graafian follicle, are evident, similar to the control mouse ovary **b**. Similarly, the testis of a cloned mouse **c** demonstrates normal spermatogenesis, as does the control mouse testis **d**. Two cloned females and males and one control female and male mice were examined.

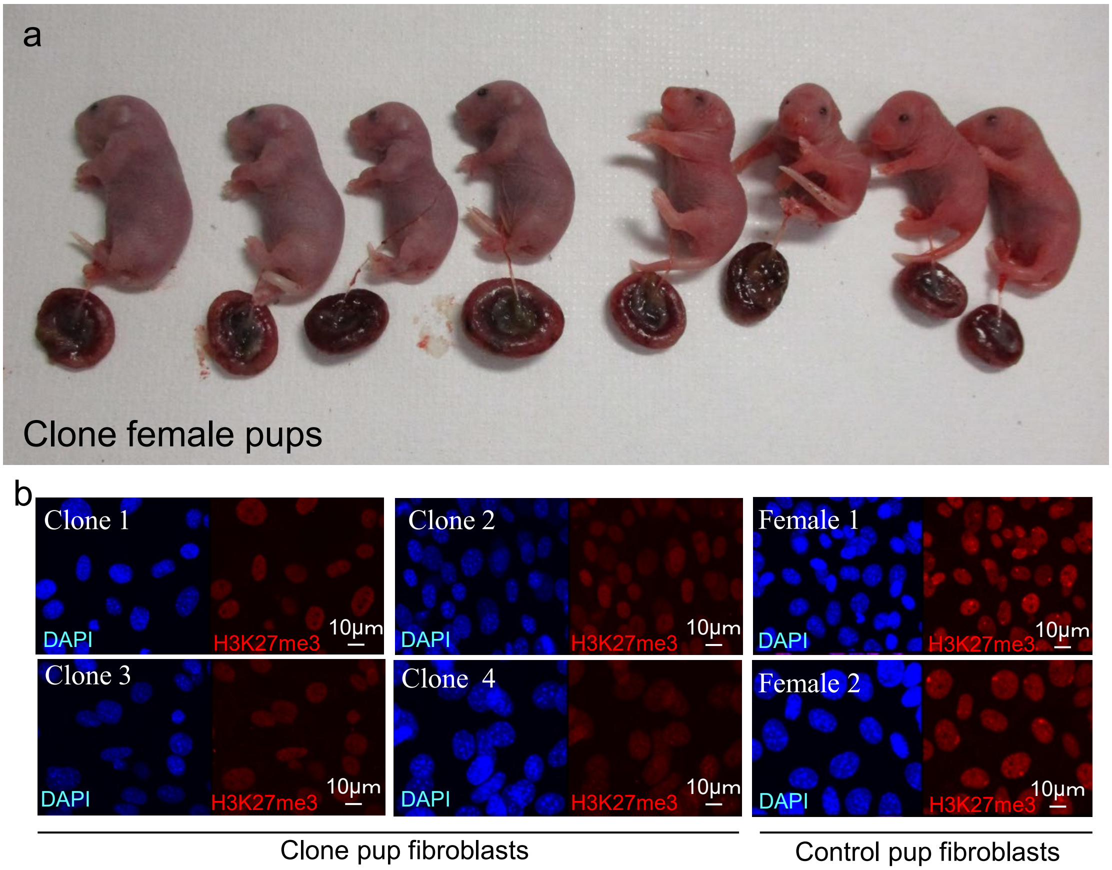


**Supplementary Fig. 5. Female cloned mice and their placenta, and number of inactive X-chromosome.**

**a,** The cloned mice were derived from male FD fibroblasts from the FDF3 ntES cell lines. Four cloned pups died owing to respiratory failure. **b**, Fibroblasts were collected from their bodies, and X-inactivation of cells were determined using anti-H3K27me3 antibodies. Four cloned female mice and two control female mice were examined.

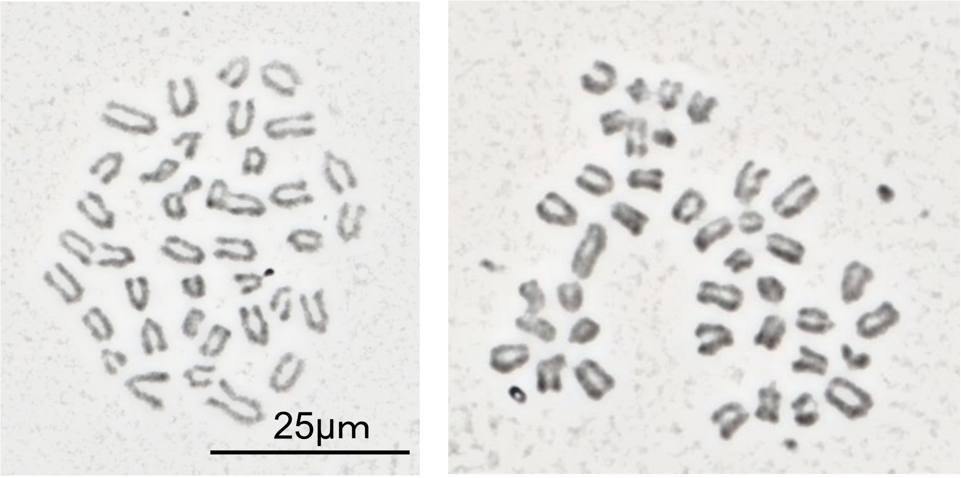


**Supplementary Fig. 6. Karyotyping of fibroblasts of female cloned mice derived from male FD fibroblasts**

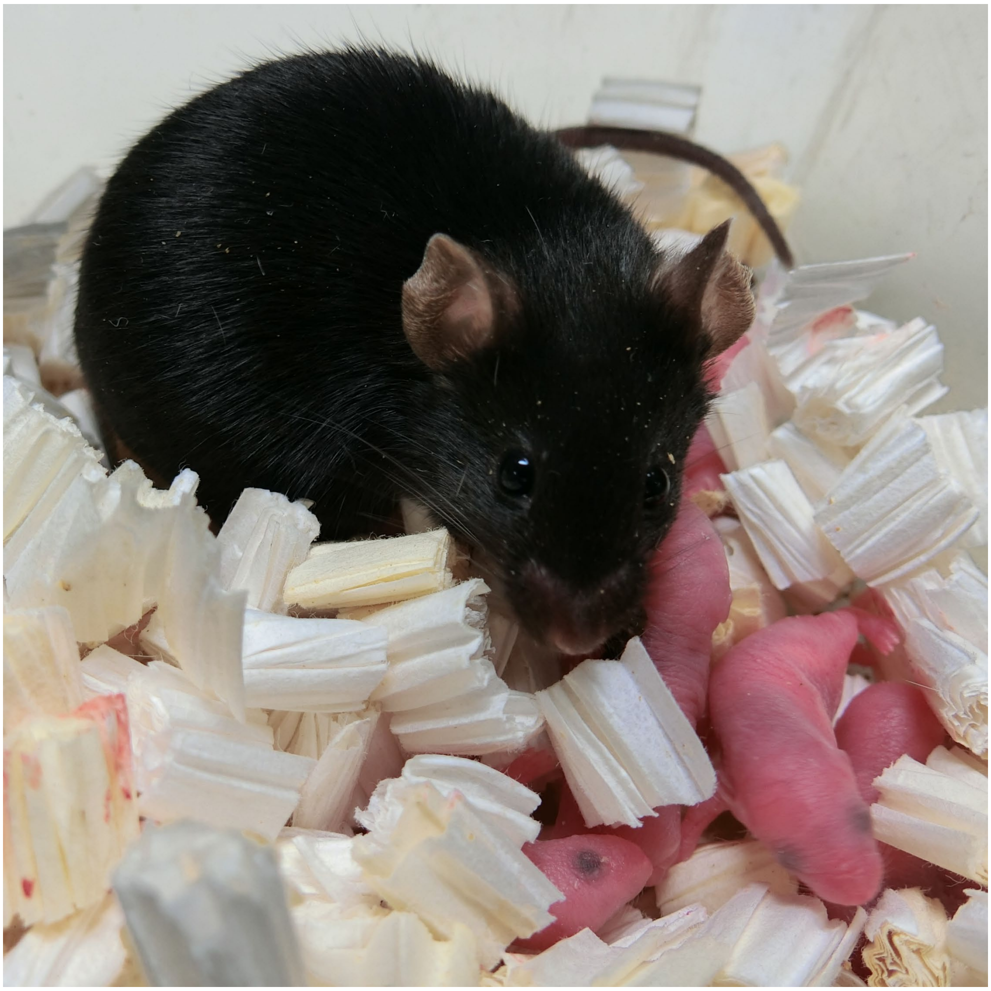

**Supplementary Fig. 7. Female cloned mouse derived from a male FD fibroblast delivered offspring after natural mating with a male.**
Cloned female mice were mated with ICR male mice naturally. Approximately 3 weeks later, the cloned mice delivered offspring, demonstrating that the cloned mice possess normal fertility.

**Supplementary Table 1. Comet DNA breakage assays of FD somatic cell nuclei**

| Condition of donor cell | Number of examined cell | Tail negative (%) | Tail positive (%) | Tail type* | |
|---|---|---|---|---|---|
| | | | | Comet (%) | Round (%) |
| Fresh | 384 | 373 (97) | 11 (3)a | 11 (100) | 0 (0) |
| FT | 165 | 1 (1) | 164 (99)b | 4 (2) | 160 (98) |
| FD-Trehalose | 161 | 36 (22) | 125 (78)b | 69 (55) | 56 (45) |
| FD-epigallocatechin | 134 | 16 (12) | 118 (88)b | 87 (74) | 31 (26) |

a vs b: $P < 0.05$ (One-way ANOVA and Tukey-multiple comparison-test).

n=3 biologically independent experiment

* The morphology of the comet tail of FD somatic cells showed two different patterns: a typical comet tail and a round shape.

**Supplementary Table 2. Timing of premature chromosome condensation (PCC) after nuclear injection into oocytes**

| | 30 min | | | | 1h | | | | 2h | | | | 3 h | | | |
|---|---|---|---|---|---|---|---|---|---|---|---|---|---|---|---|---|
| Condition of donor cell | No. exam | Intact | Early PCC | PCC | No. exam | Intact | Early PCC | PCC | No. exam | Intact | Early PCC | PCC | No. exam | Intact | Early PCC | PCC |
| Fresh control | 25 | 25 (100) | 0 | 0 | 18 | 5 (28) | 5 (28) | 8 (44) | 26 | 3 (12) | 2 (8) | 21 (80) | 22 | 0 | 0 | 22 (100) |
| Freeze-Thaw | 22 | 20 (90) | 2 (10) | 0 | 26 | 8 (31) | 14 (54) | 4 (15) | 19 | 0 | 3 (16) | 16 (84) | 22 | 1 (5) | 2 (9) | 19 (86) |
| Freeze-Dry （Trehalose） | 35 | 34 (97) | 1 (3) | 0 | 36 | 13 (36) | 15 (42) | 8 (22) | 36 | 4 (11) | 12 (33) | 20 (56) | 40 | 4 (10) | 10 (25) | 26 (65) |
| Freeze-Dry (Epigallo.) | 37 | 32 (86) | 5 (14) | 0 | 25 | 14 (56) | 9 (36) | 2 (8) | 36 | 2 (6) | 5 (14) | 29 (80) | 34 | 2 (6) | 0 | 32 (94) |

Epigallo: Epigallocatechin

n=3 biologically independent experiment

**Supplementary Table 3. Brightness of cloned pronuclei immunoassayed using anti-gamma-H2AX antibodies**

| Condition of cumulus cell | No. of assayed pseudo-pronuclear formed cloned embryo | Average brightness* of pseudo-pronucleus | Min- Max |
|---|---|---|---|
| Fresh | 27 | 1.23a | 1.06-1.58 |
| Freeze thaw | 25 | 1.24a | 1.00-1.70 |
| Freeze dry | 27 | 1.50b | 1.03-2.43 |

a vs b: $P < 0.05$ (One-way ANOVA and Tukey-multiple comparison-test).
n=3 biologically independent experiment
*The brightness of the pseudo-pronucleus was measured by Image-J, and then subtracted from the brightness of the cytoplasm.

**Supplementary Table 4. Rate of ACS of cloned two-cell embryos derived from fresh, freeze-thawed and freeze-dried somatic cells**

| Condition | Total | NCS | ACS | | | |
|---|---|---|---|---|---|---|
| | | | Light | Moderate | Heavy | Lethal |
| Fresh | 30 | 26 (86) | 1 (3) | 3 (10) | 0 (0) | 0 (0) |
| Freeze- Thaw | 43 | 23 (53) | 1 (2) | 8 (18) | 5 (11) | 6 (13) |
| Freeze- Dry | 28 | 4 (14) | 1 (3) | 4 (14) | 7 (25) | 12 (42) |

n=4 biologically independent experiment

**Supplementary Table 5. Effect of activation timing of reconstructed oocytes on the blastocyst development rate**

| Exp. | Timing of activation | No. used oocyte | No. activated oocytes | No. PN formed oocytes | No. embryo developed to | | | | | |
|---|---|---|---|---|---|---|---|---|---|---|
| | | | | | Frag. | 1-cell | 2-cell | 8-cell | Morula | Blastocyst |
| Fresh | 0.5 h | 94 | 57 | 53 | 5 | 2 | 3 | 9 | 1 | 40 (75.5) |
| | 2-3 h | 110 | 102 | 99 | 5 | 3 | 11 | 29 | 10 | 47 (47.5) |
| | 5 h | 140 | 126 | 99 | 25 | 3 | 5 | 38 | 12 | 43 (43.4) |
| FD | 0.5 h | 180 | 114 | 87 | 49 | 21 | 32 | 12 | 0 | 0 |
| | 2-3 h | 199 | 178 | 170 | 29 | 44 | 68 | 29 | 3 | 8 (4.7) |
| | 5 h | 174 | 159 | 134 | 30 | 37 | 73 | 18 | 0 | 1 (0.7) |

Embryos were cultured up to 120 h.
n=6 biologically independent experiment

**Supplementary Table 6. Cell number of trophectoderm (TE) and inner cell mass (ICM) of cloned blastocysts derived from fresh or FD somatic cells**

| Condition of donor cells | No. examined blastocyst | No. of cells | | | | |
|---|---|---|---|---|---|---|
| | | Total | Cdx2 positive | Nanog positive | Both negative | Both positive |
| Fresh | 14 | 54.9 | 41.9(76%) | 13(32%) | 3.2 | 1.9 |
| FD | 8 | 46.8 | 37.9(80%) | 10.1(30%) | 1.3 | 2.8 |

n=3 biologically independent experiment

**Supplementary Table 7. Production of cloned mice from all ntES cell lines derived from FD somatic cells**

| Line ID | Sex | Strain | Protectant | Preserv. period | No. used oocyte | No. activated oocytes | No. PN formed oocytes | No. embryos developed to 2-cell stage (/PN) | No. embryo transfer | No. offspring (/ET)[/PN] |
|---|---|---|---|---|---|---|---|---|---|---|
| FDC1 | F | BD129F1 | Treha. | 7M | 192 | 147 (76.6) | 129 (67.2) | 47 (36.4) | 47 | 0 |
| FDC2 | F | BD129F1 | | 7M | 129 | 116 (89.9) | 79 (61.2) | 44 (55.7) | 44 | 0 |
| FDC3 | F | BDF1 | | 3W | 171 | 126 (73.7) | 109 (63.7) | 67 (61.5) | 67 | 0 |
| FDC4 | F | BDF1 | | 3W | 170 | 132 (77.6) | 118 (69.4) | 66 (55.9) | 66 | 0 |
| FDC5 | F | BDF1 | | 3W | 170 | 134 (78.8) | 126 (74.1) | 74 (58.7) | 74 | 0 |
| FDC6 | F | BDF1 | | 1M | 295 | 245 (83.1) | 225 (76.3) | 127 (56.4) | 127 | 3 (2.4) (1.3) |
| FDC7 | F | BDF1 | | 1M | 94 | 92 (97.9) | 84 (89.4) | 64 (76.2) | 64 | 0 |
| FDC9 | F | BDF1 | | 1M | 410 | 379 (92.4) | 257 (62.7) | 156 (60.7) | 156 | 0 |
| FDC10 | F | BDF1 | | 1M | 153 | 125 (81.7) | 98 (64.1) | 62 (63.3) | 62 | 0 |
| FDC11 | F | BDF1 | | 5.5M | 202 | 174 (86.1) | 167 (82.7) | 98 (58.7) | 98 | 0 |
| FDC12 | F | BDF1 | | 5.5M | 205 | 172 (83.9) | 169 (82.4) | 93 (55.0) | 93 | 0 |
| FDC13 | F | BDF1 | | 1W | 293 | 228 (77.8) | 199 (67.9) | 103 (51.8) | 103 | 0 |
| FDC14 | F | BDF1 | | 1W | 259 | 231 (89.2) | 216 (83.4) | 122 (56.5) | 122 | 0 |
| FDC15 | F | BDF1 | | 9M | 217 | 197 (90.8) | 183 (84.3) | 100 (54.6) | 100 | 0 |
| FDC16 | F | BDF1 | | 9M | 310 | 280 (90.3) | 244 (78.7) | 161 (66.0) | 124 | 0 |
| FDC17G | F | 129B6G | | 2W | 168 | 141 (83.9) | 130 (77.4) | 92 (70.8) | 92 | 0 |
| FDC18G | F | 129B6G | | 2W | 132 | 108 (81.8) | 87 (65.9) | 42 (48.3) | 42 | 0 |
| FDC19G | F | 129B6G | | 2W | 176 | 158 (89.8) | 136 (77.3) | 67 (49.3) | 67 | 0 |
| FDC20G | F | 129B6G | | 2W | 86 | 67 (77.9) | 61 (70.9) | 36 (59.0) | 36 | 0 |
| FDC21G | F | 129B6G | | 2W | 88 | 67 (76.1) | 64 (72.7) | 35 (54.7) | 35 | 0 |
| FDC22 | F | BDF1 | | 1W | 197 | 180 (91.4) | 161 (81.7) | 107 (66.5) | 71 | 0 |
| FDC23 | F | BDF1 | Epigallo. | 2D | 90 | 80 (88.9) | 75 (83.3) | 41 (54.7) | 41 | 0 |
| FDC24 | F | BDF1 | | 2W | 103 | 94 (91.3) | 64 (62.1) | 34 (53.1) | 34 | 0 |
| FDC25 | F | BDF1 | | 2W | 104 | 90 (86.5) | 66 (63.5) | 26 (39.4) | 26 | 0 |
| FDC26 | F | BDF1 | | 2W | 107 | 95 (88.8) | 73 (68.2) | 40 (54.8) | 40 | 0 |
| FDC27 | F | BDF1 | | 1W | 107 | 94 (87.9) | 69 (64.5) | 36 (52.2) | 36 | 0 |
| FDC28 | F | BDF1 | | 4D | 366 | 315 (86.1) | 271 (74.0) | 141 (52.0) | 141 | 1 (0.7) (0.4) |
| FDC29 | F | BDF1 | | 4D | 169 | 147 (87.0) | 136 (80.5) | 72 (52.9) | 72 | 0 |
| FDC30 | F | BDF1 | | 1M | 152 | 144 (94.7) | 139 (91.4) | 59 (42.4) | 59 | 0 |
| FDC31 | F | BDF1 | | 2M | 151 | 131 (86.8) | 128 (84.8) | 67 (52.3) | 67 | 0 |
| FDC32 | F | BDF1 | | 3W | 459 | 403 (87.8) | 373 (81.3) | 226 (60.6) | 226 | 15 (6.6) (4.0) |
| FDC33 | F | BDF1 | | 2M | 120 | 107 (89.2) | 103 (85.8) | 51 (49.5) | 51 | 0 |
| FDC34 | F | BDF1 | | 1W | 147 | 141 (95.9) | 127 (86.4) | 83 (65.4) | 83 | 0 |
| FDC35 | F | BDF1 | | 1W | 148 | 137 (92.6) | 129 (87.2) | 71 (55.0) | 71 | 0 |
| FDC36 | F | BDF1 | | 4D | 146 | 138 (94.5) | 122 (83.6) | 69 (56.6) | 69 | 0 |
| FDF1 | M | BDF1 | Epigallo. | 2M | 118 | 112 (94.9) | 102 (86.4) | 59 (57.8) | 59 | 0 |
| FDF2 | M | BDF1 | | 2M | 118 | 110 (93.2) | 103 (87.3) | 59 (57.3) | 56 | 0 |
| FDF3 | M | BDF1 | | 2M | 1494 | 1298 (86.9) | 1136 (76.0) | 676 (59.5) | 676 | 47 (7.0) (4.1) |
| FDF4 | M | BDF1 | | 2M | 138 | 127 (92.0) | 119 (86.2) | 73 (61.3) | 73 | 0 |
| FDF5 | F | BD129F1 | | 8M | 336 | 275 (81.8) | 249 (74.1) | 114 (45.8) | 114 | 0 |
| FDF6 | M | BCF1 | | 2W | 134 | 112 (83.6) | 100 (74.6) | 49 (49.0) | 49 | 0 |
| FDF7 | M | BCF1 | | 2W | 433 | 352 (81.3) | 307 (70.9) | 183 (59.6) | 183 | 4 (2.2) (1.3) |
| FDF8 | M | BCF1 | | 2W | 444 | 381 (85.8) | 330 (74.3) | 196 (59.4) | 196 | 5 (2.6) (1.5) |
| FDF9 | M | BCF1 | | 2W | 189 | 167 (88.4) | 147 (77.8) | 92 (62.6) | 92 | 0 |
| FDF10 | F | BCF1 | | 1M | 287 | 229 (79.8) | 200 (69.7) | 145 (72.5) | 145 | 0 |
| FDF11 | F | BCF1 | | 8M | 345 | 263 (76.2) | 228 (66.1) | 141 (61.8) | 141 | 0 |
| FDF12 | F | BDF1 | | 2W | 71 | 68 (95.8) | 56 (78.9) | 40 (71.4) | 40 | 0 |
| FDF13 | F | BDF1 | | 2W | 72 | 58 (80.6) | 54 (75.0) | 30 (55.6) | 30 | 0 |
| FDF14 | F | BDF1 | | 2W | 72 | 60 (83.3) | 59 (81.9) | 36 (61.0) | 36 | 0 |

FDC8 and two ntES cell lines (before naming) derived from FD cumulus cell treated with epigallocatechin also lost accidentally.

**Supplementary Table 8. Karyotype of ntES cell lines**

| Line ID | No. of cell examined | No. of chromosomes | | | | | |
|---|---|---|---|---|---|---|---|
| | | ≤37 | 38 | 39 | 40 | 41 | 42≤ |
| FDC4 | 53 | 1 (2) | 5 (9) | 11 (21) | 29 (55) | 7 (13) | 0 |
| FDC5 | 62 | 3 (5) | 6 (10) | 10 (16) | 41 (66) | 2 (3) | 0 |
| FDC6* | 61 | 4 (7) | 3 (5) | 18 (30) | 32 (52) | 4 (7) | 0 |
| FDC17 | 48 | 2 (4) | 13 (27) | 3 (6) | 28 (58) | 1 (2) | 1 (2) |
| FDC23 | 43 | 3 (7) | 4 (9) | 10 (23) | 26 (60) | 0 | 0 |
| FDC24 | 27 | 1 (4) | 3 (11) | 9 (33) | 12 (44) | 0 | 2 (7) |
| FDC25 | 72 | 13 (18) | 9 (13) | 7 (10) | 42 (58) | 1 (1) | 0 |
| FDC26 | 45 | 8 (18) | 3 (7) | 7 (16) | 25 (56) | 2 (4) | 0 |
| FDC27 | 40 | 4 (10) | 4 (10) | 5 (13) | 26 (65) | 1 (3) | 0 |
| FDC28* | 62 | 5 (8) | 3 (5) | 11 (18) | 38 (61) | 5 (8) | 0 |
| FDC29 | 62 | 3 (5) | 3 (5) | 10 (16) | 42 (68) | 4 (6) | 0 |
| FDC30 | 85 | 9 (11) | 4 (5) | 17 (20) | 52 (61) | 2 (2) | 1 (1) |
| FDC31 | 87 | 8 (9) | 6 (7) | 10 (11) | 56 (64) | 3 (3) | 4 (5) |
| FDC32* | 61 | 19 (31) | 4 (7) | 13 (21) | 23 (38) | 0 | 2 (3) |
| FDC33 | 80 | 8 (10) | 12 (15) | 17 (21) | 41 (51) | 1 (1) | 1 (1) |
| FDC34 | 47 | 7 (15) | 2 (4) | 7 (15) | 29 (62) | 2 (4) | 0 |
| FDC35 | 25 | 3 (12) | 3 (12) | 3 (12) | 16 (64) | 0 | 0 |
| FDC36 | 47 | 3 (6) | 3 (6) | 12 (26) | 29 (62) | 0 | 0 |
| FDF1 | 103 | 4 (4) | 2 (2) | 11 (11) | 59 (57) | 24 (23) | 3 (3) |
| FDF2 | 97 | 4 (4) | 5 (5) | 19 (20) | 64 (66) | 4 (4) | 1 (1) |
| FDF3* | 74 | 8 (11) | 14 (19) | 46 (62) | 6 (8) | 0 | 0 |
| FDF4 | 38 | 0 | 2 (5) | 6 (16) | 29 (76) | 1 (3) | 0 |
| FDF5 | 46 | 6 (13) | 7 (15) | 32 (70) | 1 (2) | 0 | 0 |
| FDF6 | 16 | 0 | 0 | 4 (25) | 11 (69) | 0 | 1 (6) |
| FDF7* | 41 | 6 (15) | 3 (7) | 6 (15) | 23 (56) | 3 (7) | 0 |
| FDF8* | 46 | 5 (11) | 1 (2) | 6 (13) | 31 (67) | 3 (7) | 0 |
| FDF9 | 40 | 2 (5) | 5 (13) | 11 (28) | 20 (50) | 1 (3) | 1 (3) |
| FDF10 | 30 | 3 (10) | 0 | 8 (27) | 19 (63) | 0 | 0 |
| FDF12 | 55 | 4 (7) | 1 (2) | 9 (16) | 37 (67) | 3 (5) | 1 (2) |
| FDF13 | 52 | 4 (8) | 4 (8) | 8 (15) | 36 (69) | 0 | 0 |

* Cloned mice were born from these cell lines

**Supplementary Table 9. Body and placental weight of cloned mice derived from FD somatic cells**

| Generation | No. of cloned mice | Body weight | | | | Placental weight | | | |
|---|---|---|---|---|---|---|---|---|---|
| | | Average | SD | Min | Max | Average | SD | Min | Max |
| FDC6 | 3 | 1.80 | 0.33 | 1.48 | 2.13 | 0.30 | 0.08 | 0.26 | 0.39 |
| FDC28 | 1 | 3.06 | - | - | - | 0.57 | - | - | - |
| FDC32 | 15 | 1.88 | 0.26 | 1.40 | 2.26 | 0.35 | 0.14 | 0.20 | 0.78 |
| FDF3 | 47 | 1.82 | 0.37 | 0.96 | 2.52 | 0.35 | 0.15 | 0.14 | 0.79 |
| FDF7 | 4 | 1.89 | 0.74 | 0.90 | 2.52 | 0.31 | 0.14 | 0.12 | 0.44 |
| FDF8 | 5 | 2.03 | 0.47 | 1.27 | 2.45 | 0.45 | 0.13 | 0.30 | 0.54 |
| Total | 42 | 1.86 | 0.43 | 0.90 | 3.06 | 0.37 | 0.15 | 0.12 | 0.79 |

**Supplementary Table 10. Fertility of cloned mice**

| Donor cell type | Sex of donor mouse | ID of ntES cell line | ID of Cloned mouse | Sex of cloned mouse | Fertility* |
|---|---|---|---|---|---|
| Cumulus | F | FDC6 | C6-1 | F | Yes |
| | | | C6-2 | F | Yes |
| | | FDC32 | C32-1 | F | Yes |
| Fibroblast | M | FDF3 | F3-1 | F | Yes |
| | | | F3-2 | F | Yes |
| | | | F3-3 | F | Yes |
| | | | F3-4 | F | Yes |
| | | | F3-5 | F | Yes |
| | | | F3-6 | F | Yes |
| Fibroblast | M | FDF7 | F7-1 | M | Yes |
| | | FDF8 | F8-1 | M | Yes |
| | | | F8-2 | M | Yes |

*Fertility was confirmed when clone or mating partner deliver the offspring.

**Supplementary Table 11. Karyotype of cloned mice derived from the FDF3 ntES cell line**

| Line ID | No. of cell examined | No. of chromosomes | | | | | |
|---|---|---|---|---|---|---|---|
| | | ≤37 | 38 | 39 | 40 | 41 | 42≤ |
| FDF3pup1 | 10 | 3 (30) | 1 (10) | 6 (60) | 0 | 0 | 0 |
| FDF3pup2 | 49 | 4 (8) | 9 (18) | 32 (65) | 4 (8) | 0 | 0 |
| FDF3pup3 | 26 | 4 (15) | 2 (8) | 15 (58) | 4 (15) | 1 (4) | 0 |
| FDF3pup4 | 9 | 2 (22) | 5 (56) | 2 (22) | 0 | 0 | 0 |

**Supplementary Table 12. Production of cloned mice from guaranteed ntES cell lines**

| ID of ntES cell line | Donor cell type | Sex | Chromosome integrity of cloned mice | Mouse strain | No. used oocyte | No. PN formed oocytes | No. embryos developed to 2-cell stage (%) | No. offspring (%/ET) [%/PN]* |
|---|---|---|---|---|---|---|---|---|
| FDC6 | Cumulus | F | Intact | BDF1 | 295 | 225 | 127 | 3 (2.4) [1.3] |
| FDC28 | | | Intact | BDF1 | 366 | 271 | 141 | 1 (0.7) [0.4] |
| FDC32 | | | Intact | BDF1 | 459 | 373 | 226 | 15 (6.6) [4.0] |
| FDF3 | Fibroblast | M | Y chromosome lost | BDF1 | 1494 | 1298 | 676 | 47 (7.0) [4.1] |
| FDF7 | | | Intact | BCF1 | 433 | 307 | 183 | 4 (2.2) [1.3] |
| FDF8 | | | Intact | BCF1 | 444 | 330 | 196 | 5 (2.6) [1.5] |
| Total | | | | | 3491 | 2804 | 1549 | 75 (4.8) [2.7] |

* There is no significant difference between all groups
