# Supplementary material for: Healthy cloned offspring derived from freeze-dried somatic cells
Source: Nat Commun. 2022 Jul 5;13:3666. doi: 10.1038/s41467-022-31216-4 (PMC9256722; doi:10.1038/s41467-022-31216-4)
Supplement: Supplementary file 3 — Reporting Summary [file 41467_2022_31216_MOESM3_ESM.pdf]

nature portfolio

Corresponding author(s): NCOMMS-22-07506-T

Last updated by author(s): Apr 22, 2022

Reporting Summary

Nature Portfolio wishes to improve the reproducibility of the work that we publish. This form provides structure for consistency and transparency in reporting. For further information on Nature Portfolio policies, see our Editorial Policies and the Editorial Policy Checklist.

Statistics

For all statistical analyses, confirm that the following items are present in the figure legend, table legend, main text, or Methods section.

n/a	Confirmed	
☐	☒	The exact sample size (n) for each experimental group/condition, given as a discrete number and unit of measurement
☐	☒	A statement on whether measurements were taken from distinct samples or whether the same sample was measured repeatedly
☐	☒	The statistical test(s) used AND whether they are one- or two-sided<br>*Only common tests should be described solely by name; describe more complex techniques in the Methods section.*
☐	☒	A description of all covariates tested
☐	☒	A description of any assumptions or corrections, such as tests of normality and adjustment for multiple comparisons
☐	☒	A full description of the statistical parameters including central tendency (e.g. means) or other basic estimates (e.g. regression coefficient) AND variation (e.g. standard deviation) or associated estimates of uncertainty (e.g. confidence intervals)
☒	☐	For null hypothesis testing, the test statistic (e.g. F, t, r) with confidence intervals, effect sizes, degrees of freedom and P value noted<br>*Give P values as exact values whenever suitable.*
☒	☐	For Bayesian analysis, information on the choice of priors and Markov chain Monte Carlo settings
☒	☐	For hierarchical and complex designs, identification of the appropriate level for tests and full reporting of outcomes
☒	☐	Estimates of effect sizes (e.g. Cohen's d, Pearson's r), indicating how they were calculated

Our web collection on statistics for biologists contains articles on many of the points above.

Software and code

Policy information about availability of computer code

Data collection	n/a because only wet experiments
Data analysis	Prism, GraphPad Software

For manuscripts utilizing custom algorithms or software that are central to the research but not yet described in published literature, software must be made available to editors and reviewers. We strongly encourage code deposition in a community repository (e.g. GitHub). See the Nature Portfolio guidelines for submitting code & software for further information.

Data

Policy information about availability of data

All manuscripts must include a data availability statement. This statement should provide the following information, where applicable:

- Accession codes, unique identifiers, or web links for publicly available datasets
- A description of any restrictions on data availability
- For clinical datasets or third party data, please ensure that the statement adheres to our policy

All data were provided as supplementary table and Excel file. There is no NGS analysis.

Field-specific reporting

Please select the one below that is the best fit for your research. If you are not sure, read the appropriate sections before making your selection.

☒ Life sciences ☐ Behavioural & social sciences ☐ Ecological, evolutionary & environmental sciences

For a reference copy of the document with all sections, see nature.com/documents/nr-reporting-summary-flat.pdf

Life sciences study design

All studies must disclose on these points even when the disclosure is negative.

Sample size	We have repeated this experiment using 6 mouse strains two different tissue and both sex. We used total 18867 oocytes.
Data exclusions	All data were provided
Replication	We succeeded to produce many cloned mice from 6 independent donor mice
Randomization	We used inbred or F1 mouse strain and randomly pick upped donor mice from them.
Blinding	This study did not compare between group

Reporting for specific materials, systems and methods

We require information from authors about some types of materials, experimental systems and methods used in many studies. Here, indicate whether each material, system or method listed is relevant to your study. If you are not sure if a list item applies to your research, read the appropriate section before selecting a response.

Materials & experimental systems

n/a	Involved in the study	
☐	☒	Antibodies
☒	☐	Eukaryotic cell lines
☒	☐	Palaeontology and archaeology
☐	☒	Animals and other organisms
☒	☐	Human research participants
☒	☐	Clinical data
☒	☐	Dual use research of concern

Methods

n/a	Involved in the study	
☒	☐	ChIP-seq
☒	☐	Flow cytometry
☒	☐	MRI-based neuroimaging

Antibodies

Antibodies used

anti-beta-tubulin mouse monoclonal antibody labelled with FITC (1:1000; Sigma-aldrich, F2043)
anti-phospho-H2AX (Ser139) rabbit polyclonal antibody (1:500; Millipore-Merck, Darmstadt, Germany, 07-164)
an anti-histone H3 (dimethyl K9) mouse monoclonal antibody (1:500, Abcam, Cambridge, UK, ab1220).
an anti-CDX2 mouse monoclonal antibody (1:500; BioGenex, San Ramon, CA, USA, MU392A-UC)
an anti-Nanog rabbit polyclonal antibody (1:500; Abcam, Cambridge, UK, ab80892)
anti-H3K27me3 rabbit polyclonal antibodies (1:500; Millipore-Merck, Darmstadt, Germany 07-449)

Validation

anti-beta-tubulin mouse FITC antibody produced in mouse (1:1000;sima F2043)
https://www.sigmaaldrich.com/JP/en/product/sigma/f2043?
gclid=Cj0KCQjwgYSTBhDKARIsAB8KuksCMbMcv3WdGAo9RBtMmd7JmAE7HXguhbJ6FD2Tff4c3xr9zW3c7zUaAtjNEALw_wcB

anti-phospho-H2AX (Ser139) rabbit polyclonal antibody (1:500; Millipore-Merck,07-164)
https://www.sigmaaldrich.com/JP/ja/product/mm/07164

an anti-histone H3 (dimethyl K9) mouse monoclonal antibody (1:500, Abcam, Cambridge, UK ab1220).
https://www.abcam.co.jp/histone-h3-di-methyl-k9-antibody-mabcam-1220-chip-grade-ab1220.html

an anti-CDX2 mouse monoclonal antibody (1:500; BioGenex, San Ramon, CA, USA, MU392A-UC)
http://store.biogenex.com/us/applications/ihc/controls/controls/anti-cdx-2-clone-cdx2-88.html

an anti-Nanog rabbit polyclonal antibody (1:500; Abcam, Cambridge, UK, ab80892)
https://www.abcam.co.jp/nanog-antibody-ab80892.html

anti-H3K27me3 rabbit polyclonal antibodies (1:500; Millipore-Merck, Darmstadt, Germany 07-449)

https://www.sigmaaldrich.com/JP/ja/product/mm/07449

Animals and other organisms

Policy information about studies involving animals; ARRIVE guidelines recommended for reporting animal research

Laboratory animals	mouse
Wild animals	n/a
Field-collected samples	n/a
Ethics oversight	All animal experiments were conducted in accordance with the Guide for the Care and Use of Laboratory Animals and were approved by the Institutional Committee of Laboratory Animal Experimentation of the University of Yamanashi (reference number: A29–24), which followed the ARRIVE guidelines.

Note that full information on the approval of the study protocol must also be provided in the manuscript.
